# Advancing leadership in surgery: a realist review of interventions and strategies to promote evidence-based leadership in healthcare

**DOI:** 10.1186/s13012-023-01274-3

**Published:** 2023-05-13

**Authors:** Julia Gauly, Rachel Court, Graeme Currie, Kate Seers, Aileen Clarke, Andy Metcalfe, Anna Wilson, Matthew Hazell, Amy Louise Grove

**Affiliations:** 1 Division of Health Sciences, Warwick Medical School, University of Warwick, Coventry, CV4 7HL UK; 2 Warwick Business School, University of Warwick, Scarman Rd, Coventry, CV4 7AL UK; 3 Warwick Clinical Trials Unit, Warwick Medical School, University of Warwick, Gibbet Hill Campus, Coventry, CV4 7AL UK

**Keywords:** Leadership, Reaslist review, Organisational leadership interventions, Surgery

## Abstract

**Background:**

Healthcare systems invest in leadership development of surgeons, surgical trainees, and teams. However, there is no agreement on how interventions should be designed, or what components they must contain to be successful. The objective of this realist review was to generate a programme theory explaining in which context and for whom surgical leadership interventions work and why.

**Methods:**

Five databases were systematically searched, and articles screened against inclusion considering their relevance. Context-mechanism-outcome configurations (CMOCs) and fragments of CMOCs were identified. Gaps in the CMOCs were filled through deliberation with the research team and stakeholder feedback. We identified patterns between CMOCs and causal relationships to create a programme theory.

**Results:**

Thirty-three studies were included and 19 CMOCs were developed. Findings suggests that interventions for surgeons and surgical teams improve leadership if timely feedback is delivered on multiple occasions and by trusted and respected people. Negative feedback is best provided privately. Feedback from senior-to-junior or peer-to-peer should be delivered directly, whereas feedback from junior-to-senior is preferred when delivered anonymously. Leadership interventions were shown to be most effective for those with awareness of the importance of leadership, those with confidence in their technical surgical skills, and those with identified leadership deficits. For interventions to improve leadership in surgery, they need to be delivered in an intimate learning environment, consider implementing a speak-up culture, provide a variety of interactive learning activities, show a genuine investment in the intervention, and be customised to the needs of surgeons. Leadership of surgical teams can be best developed by enabling surgical teams to train together.

**Conclusions:**

The programme theory provides evidence-based guidance for those who are designing, developing and implementing leadership interventions in surgery. Adopting the recommendations will help to ensure interventions are acceptable to the surgical community and successful in improving surgical leadership.

**Trial registration:**

The review protocol is registered with PROSPERO (CRD42021230709).

**Supplementary Information:**

The online version contains supplementary material available at 10.1186/s13012-023-01274-3.

Contributions to the literature

- The paper provide the first theoretically informed realist review of the scientific evidence-base for leadership development interventions used in surgery.
- We provide a novel programme theory to explain in which context and for whom, leadership interventions in surgery work and why.
- The realist review findings provides evidence-based guidance for those who are designing, planning, and implementing evidence-based leadership interventions in surgery.

## Background

Investment in leadership development in healthcare is substantial. Reports indicate an estimated annual spend on leadership development in the USA at $50 billion (USD) [1, 2]. In 2019, the National Health Service (NHS) in England invested £2 million to help boost leadership development [3] and made individual leadership and capability development fundamental to delivery of the NHS Long-Term Plan [4]. Over the last three decades, we have seen a growing trend in health systems around the world placing great emphasis and resource on improving ‘clinical leadership’ [5]. Traditionally, clinical leadership encompassed leadership delivered by doctors and nurses [6]. More recently, clinical leadership has expanded to comprise anyone trained to deliver frontline care [6].

In the context of surgery, the surgical profession has increasingly recognised the need for high quality leadership both in and outside of the operating theatre [7, 8]. For example, the Royal College of Surgeons of England recommend that consultant surgeons have the responsibility to develop an effective team through leadership and teambuilding [7]. The necessity for surgeons and surgical teams to lead, inspire, and manage a team to meet the needs of patients, however, is not a substantial, or evidence-based component of the surgical curriculum.

In the UK, the Department for Health and Social Care (DHSC) recently called for more inclusive leadership in the healthcare professions, which aims to adopt a collaborative approach to leadership practice [9]. However, evidence for endorsing this approach was lacking. It was not clear how inclusive collaborative leadership could or should be achieved in practice. Within healthcare literature, the focus tends towards leadership development which advances the leadership skills of individuals–not collective teams [10]. This skills-based approach, promoted by Mumford and colleagues (2007), categorises leadership into types of skills, for example, cognitive, interpersonal, business, and strategic skills [10]. NHS England, alternatively, describe leadership development as confidence building, understanding practical levers, widening perspectives, and talent management [6]. Whilst these are more generic terms, they are still person centric. Nonetheless, these individualist approaches target the ability and motivation of surgeons to improve their own leadership. Little attention is given to the multidisciplinary teams, organisation, and environmental contexts in which leadership plays out in. Skills-based leadership, therefore, fails to account for the contextual opportunities which enable leadership to be enacted in the inherently social conditions of the healthcare sector.

In contrast, Ability, Motivation, and Opportunity (AMO) theory describes how the interplay between ability, motivation, and opportunity (of a person, a team, or a department) to enact leadership, gives us a measure of an leadership performance and performance-related outcomes [11]. This enables us to embrace leadership as a distributed and collective process [12]. In the distributed leadership literature, leadership becomes a shared process across a collective group, where people have common organisational perspectives, goals, and shared actions [12]. Framing leadership in this way allows us to move away from traditional heroic leadership tropes, to recognise the contribution that groups of people, such as a surgical team, make to leadership processes and practices [13].

Despite the significant investment in healthcare leadership development, and the numerous systematic reviews which have been conducted to determine the effectiveness of specific leadership interventions (such as team-training and co-leadership) [14–19], there is no agreement on what surgical leadership is, what leadership capabilities are, or how we can ensure they are developed and implemented effectively. Most important for the healthcare sector is that leadership development is viewed as a workforce intervention, and if funded by public money, should be underpinned by a rigorous evidence-base. Therefore, it is vital that workforce interventions, aimed to improve leadership processes, are appropriate and able to achieve effective outcomes. Not only to justify the significant expenditure, but to ensure that advanced leadership can improve the quality of patient care. This is a challenge in many areas of healthcare delivery, including surgery [20].

We aimed to fill this gap by conducting a realist review of interventions and strategies to promote evidence-based leadership in healthcare. The goal of our review, is to develop a programme theory to answer the following question: In which context and for whom, can interventions and strategies improve the leadership of surgical trainees, surgeons, and surgical teams and why?

## Methods

### Realist review approach

Realist reviews are a theory-based approach to synthesising existing evidence. This review follows the Realist and Meta-narrative Evidence Syntheses: Evolving Standards (RAMESES) quality and reporting standards [21] which involves a process of focusing the review, developing programme theory, developing a search strategy, selection, and appraisal of documents, and applying realist principles to the analysis of data. We have provided a flow diagram which details the review process, and how the RAMESES standards were followed (see Additional file 1) [21].

According to realist philosophy, interventions that are context-dependent, and those that are successful in certain contexts but not in others, can be described as complex. Leadership development is an inherently complex intervention [22], realist review methods enable us to unpack the ‘black box’ of leadership. Realist reviews aim to develop a programme theory, which is grounded in existing literature, that seeks to explain why and how complex interventions work. They identify the underlying mechanisms (the hidden actions) that are triggered in certain contexts, which lead to specific outcomes [23]. These causal chains are referred to as Context-Mechanism-Outcome configurations (CMOCs). In our review, we have adopted definitions of context, mechanisms, and outcomes as used by Wong and colleagues [21]. They describe context as the “backdrop of programs and research” and the condition that “triggers and/or modifies the behavior of a mechanism” [24]. A mechanism then, is the agent of change, the “underlying entities, processes, or structures which operate in particular contexts to generate outcomes of interest” [25]. Because mechanisms are underlying, they can be difficult to identify in the literature. Finally, the outcome is the entity that changes as a consequence of the context, triggering the mechanism.

### Focusing the review

The review scope was developed iteratively through a scoping search of the literature, multiple expert stakeholder consultations and discussion between the research team. Experts included orthopaedic surgeons at various career stages and surgical trainees, an orthopaedic Training Programme Director, members of a orthopaedic leadership Action Learning Set and academics with expertise in clinical leadership. We developed the study protocol which was registered with PROSPERO CRD42021230709 and published [26]. To inform our initial programme theory, we searched for theoretical papers on surgical leadership. Our information specialist (RC) designed and conducted a systematic search in five databases not limited by date.

The theory search identified 8382 articles. Two authors independently screened the titles and abstracts identified in one database search (MEDLINE *n* = 4012). Included papers were obtained at full text and read for relevance to the review. Potentially relevant articles were summarised and discussed with the wider project team. During this process it became apparent that the theoretical papers were unhelpful in progressing our initial programme theory. Many articles appeared generic, describing the importance of ‘good’ leadership at different levels (i.e. macro, meso, micro) of healthcare, but not specifically how leadership should be conceptualised, or which component parts could form the basis of future interventional research. Following discussion with experts we therefore, ceased this theoretical scoping activity and focused our resources on the identification of empirical studies, which we considered more informative for narrowing the scope of our initial programme theory.

### Developing programme theory

Following scoping, our initial programme theory included leadership development outcomes for individual surgeon leadership, patient outcomes and organisational outcomes [26]. After discussions with expert stakeholders and the research team, we narrowed the scope to focus only on outcomes for surgeons, surgical teams, and trainees. That is those professional groups or communities of individuals who deliver surgical services. Our initial programme theory depicted organisational and patient outcomes as distal outcomes. These were the outcomes which may (or may not) develop as a result of improvements to the professional group—i.e. the proximal outcome of the leadership intervention (see Additional file 1). However, this large scope generated an intractable volume of literature and diluted the causal links between the intervention and intended outcome. A narrowed scope on proximal outcomes enabled us to meaningfully categorise the interventions and strategies used to promote evidence-based leadership in healthcare.

### Developing a search strategy

A systematic search strategy for empirical studies was developed in MEDLINE (Ovid) by our information specialist (RC). The search was conducted in July 2021 and adapted to a variety of bibliographic databases relevant to the scope of the review (MEDLINE (Ovid), Embase (Ovid), PsycINFO (Ovid), Cochrane Library (Wiley), HMIC (Ovid), Abi/INFORM Global (Proquest).

A range of relevant search terms were included, combining the concepts of leadership interventions with surgeon/surgical team. The empirical search was limited to literature published in English after 2014, the year in which the ‘*Surgical Leadership: A guide to best practice*’ guidance was first published by the Royal College of Surgeons of England (an updated 2018 version has since been published) [8]. An example of the search strategy in MEDLINE (Ovid) is provided (see Additional file 1). The references of all included documents and relevant reviews (systematic and narrative) were dual screened to identify further relevant documents for consideration.

### Selection and appraisal of documents

Studies were screened at title and abstract stage against the inclusion criteria listed in Table 1.

**Table 1:** Inclusion criteria

• Empirical studies of any study design published after 2014 in English
• Studies that focus on any type of intervention that are put in place to improve [as defined in the publication] the leadership of surgeons, surgical trainees and surgical teams (e.g., mentoring, coaching, simulation training, taught courses, etc.)
• Studies including surgeons of any training level in settings including hospitals, clinics, academic organisations, and external training settings
• Studies that report results for surgeons separately from other study populations

Two reviewer pairs independently screened all titles and abstracts identified through the empirical search (AW + MS, AG + MH). Full text articles were obtained and screened independently by two reviewers (MH + AW). Full-text articles were screened against the inclusion criteria and with consideration to their relevance [23, 27].

Disagreements were resolved through discussion including a third reviewer (AG or JG). Two reviewer pairs (AW + MH) independently screened the reference lists of identified reviews and all included studies to identify relevant articles.

### Applying realist principles to the analysis

#### Data extraction

A data extraction template was piloted (JG) by the research team members (AG, AW, MH) and minor adaptations were made. Data was extracted by one research team member (MH or AW) and checked by one research team members (JG). We abstracted all data from the study that might be relevant to the research question into one document per study for review by the team [28]. Extracted data could include descriptive information such as geography and participants, but also data which could inform CMOCs or fragments of CMOCs. Data extracted are listed in Table 2.

**Table 2:** Data extracted from studies

• Year of publication
• Country
• Study design
• Population
• Intervention
• Setting
• Population
• All textual data relevant to the research quesiton

#### Appraisal of the evidence

Relevance for purpose was the most important factor in determining relevance for inclusion in our review and articles were not be excluded based on their quality [27]. Nevertheless, since understanding of rigour is relevant for our synthesis and for understanding the strength of our findings [21], we used the mixed methods appraisal tool (MMAT) to review the quality of included studies [29]. All included articles were assessed by one reviewer (AW or MH) and 25% of studies were checked by a second reviewer (JG). Disagreements were resolved by a third reviewer (JG). We selected the MMAT as it can be used for all study designs [29]. We grouped articles into low, medium, or high quality [30, 31].

#### Data synthesis

All studies relevant to the individual surgeon leadership were grouped into the four skill categories suggested by Mumford et al. [10]. For example, interventions which included one to one mentorship or coaching were grouped as interpersonal skills.

Discussion between the research team and expert stakeholders generated additional categories of leadership intervention where the focus was broader, for example team-based simulations. Next, the first author (JG) identified CMOCs and CMO fragments (e.g., Context and Outcome; Context and Mechanism) which were copied into a separate word document for each type of leadership intervention (mentoring, coaching, simulation training, leadership course, feedback intervention, and debriefing) for discussion with the wider team. An excerpt from each article was selected as supporting evidence of the CMOC or CMO fragment. Next, we reviewed, compared, and contrasted all CMOCs and CMO fragments and tried to identify patterns and causal relationships between them.

We synthesised CMOCs and CMO fragments iteratively through verbal discussion, reading and commenting on each other’s configurations. We documented the CMOCs and CMO fragments in Word documents and in a programme theory diagram. We refined the diagram through discussion with the research team members. In several CMOCs, a mechanism or mechanisms were not immediately obvious, and not directly referred to in the literature. In these cases, the review team would suggest mechanisms that offered a potential ‘fit’ with the data [28]. We took a pragmatic approach and consulted expert stakeholders regularly to review and refine our CMOCs and programme theory and fill any gaps in our theory [28]. Where parts of a CMOC or CMO fragment were not grounded in the literature or expert stakeholders’ experience, this is clearly highlighted in our findings to enhance transparency.

### Stakeholder consultations

A group of expert stakeholders was identified through the established network of the research team members and included two senior academics, four consultant surgeons and one surgical trainee. Stakeholders’ feedback on the consistency and plausibility of CMOCs and the programme theory was discussed in virtual meetings and incorporated into the final programme theory.

## Results

**Fig. 1:**
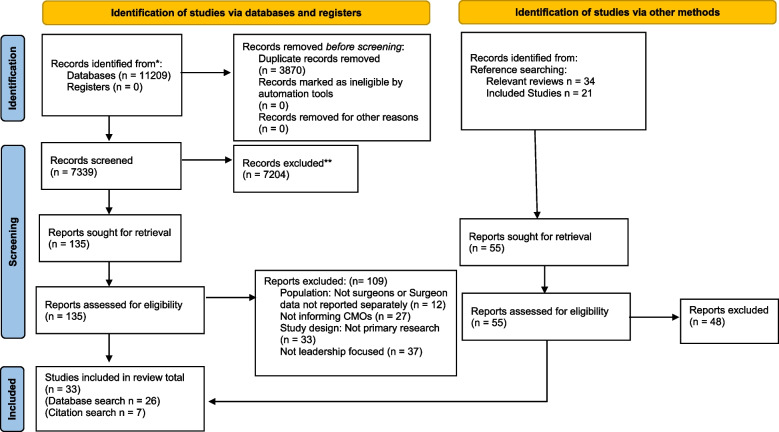
Overview of search results

Thirty-three articles were included in our review [32–64] (see Fig. 1). An overview of the characteristics of included studies is provided in Table 3. Studies included in our realist review were judged on their rigour, i.e. whether we considered the reported method used to generate the piece of data credible and/or trustworthy [21]. The overall quality of included studies varied: 21 studies were rated as high, eight studies as medium and four as low quality (see Table 3).

**Table 3:** Characteristics of included studies

Study	Country	Study type	Participants (number of surgeons)	Specialty	Setting	Leadership Intervention of interest	Quality
Ahmed 2019 [32]	Pakistan	Mixed methods RCT including non-technical skills scale followed by focus group	28	Obstetrics and gynaecology	Hospital operating theatre	Non-technical skills training (didactic and practical sessions)	Medium
Al-Jundi 2016 [33]	UK	Mixed methods-survey with open-ended questions and Non-technical skills scale	76	Not specified	1 teaching and 2 district general hospitals (NHS)	Non-technical skills training (acceptability of assessments)	Hig
Brindle 2018 [34]	USA	Qualitative–semi-structure interviews	4	Surgical quality improvement implementation leaders	3 large public health systems (20 hospitals) and one army medical centre	Surgical debriefing program	High
Brook 2020 [35]	USA	Quantitative survey	117	Academic orthopaedic surgeons	Academic residency programs	Mentoring	Low
Cochran 2019 [36]	USA	Qualitative interviews	15	Academic surgeons (varying specialties)	Unclear	Mentoring	High
Dominguez 2021 [37]	Colombia	Qualitative-semi structured interviews	16	Surgeons identified as exponents of a transformational leadership style	unclear	‘Job crafting’ from senior surgeons to enhance residents	High
Doumouras 2017 [38]	Canada	Quantitative non-randomised prospective study including non-technical skills scale	23	General surgery residents	McMaster University General Surgery Residency Program	Non-technical skills training (crisis simulation training with debrief based on crisis resource management principles)	High
Foley 2021 [39]	Australia	Quantitative survey	118	Consultant surgeons (varying specialities)	2 tertiary hospitals	Peer-based coaching	High
Gostlow 2017 [40]	Australia	Quantitative case–control including non-technical skills scale	70	Surgical trainees (40), experienced consultants (30) (varying specialties mainly general surgery)	Unclear	Non-technical skills training (before and after the introductions of Royal Australasian College of Surgeons Surgical Education and Training (SET) curriculum)	High
Greenberg 2018 [41]	USA	Mixed methods survey, focus groups and interview	12	Varied experience and specialty	Range- academic, private urban and private rural	Video based coaching	Medium
Gregory 2018 [42]	USA	Quantitative retrospective including 360° feedback	12	Orthopaedic surgery service chiefs	urban academic orthopedic surgery department	360° feedback program and coaching	High
Hart 2020 [43]	USA	Quantitative survey	149	Orthopaedic residents	University, community and military-based programs	Mentoring	Low
Hill 2018 [44]	USA	Quantitative survey	17	Junior residents general surgery	University hospital	Leadership development programme (lectures with discussion)	Medium
Hu J 2020 [45]	USA and Canada	Quantitative non-randomised study including 360° feedback	547	Variety of specialties—neurosurgery, orthopaedic, obstetrics and gynaecology, cardiothoracic, plastics, urology	Academic and community hospitals	360° feedback programme (Group 1-Feedback report only Group 2-Debriefing only Group 3-Debriefing and coaching)	High
Hu Y 2016 [46]	USA	Quantitative study (video-based observational)	5	General and oncological surgery	Quaternary care hospital	Assessment of leadership styles in the operating room	Medium
Jaffe 2016 [47]	USA	Qualitative interview	24	Academic surgeons (variety of specialities) Many in leadership positions—section and division heads and program directors	University	Views on leadership development programme	High
Jayasuria-Illesinghe 2016 [48]	Sri Lanka	Qualitative interview	15	Junior and senior general surgeons	Teaching hospital	Views on teamwork, team member roles, and team processes	High
Kaderli 2015 [49]	Switzerland	Quantitative survey	512	General surgeons—junior and senior	Mixed-university hospital, referral, and regional hospitals and private practice	Mentoring	Low
Kawase 2016 [50]	Japan, USA, Finland, and Hong Kong	Quantitative survey	225	Women surgeons (varied specialties and career stages)	60% were affiliated with an academic institution, 22% a general hospital, 12% private practice	Views on factors that promote or impede advancement of women as leaders	Medium
Lee SH 2021 [51]	USA	Quantitative prospective pre-post cohort study	36	Orthopaedic surgeons-attending, resident and fellow level	Hospital	Leadership development programme (communication, lectures, discussion, and grand rounds)	Low
Lee T 2020 [52]	USA	Quantitative survey	52	American College of Surgeons Society of Surgical Chairs	Academic Surgery Departments	Experience of leadership and academic advancement	Medium
Mutabdzic 2015 [53]	Canada	Qualitative interview	14	Staff surgeons -Varied specialties	University affiliated hospitals	Coaching	High
Nicksa 2015 [54]	USA	Quantitative pre- and post-evaluation and survey feedback	37	Junior surgical residents (post-graduate year 1 and 2) on general, vascular and cardiothoracic surgery	San Francisco VA Medical Center	Simulation	High
Nurudeen 2015 [55]	USA	Quantitative feedback survey including 360° feedback	118	Attending surgeons cardiac, thoracic, vascular, orthopedic, plastic, and general surgery departments	8 hospitals including academic medical centers and university affiliated community hospitals with a voluntary medical staff	360° feedback programme	Medium
Pena 2015 [56]	Australia	Quantitative single-blinded, prospective comparative trial including non-technical skills scale	40	Trainees and fellows of Mixed specialty's	Unclear	Simulation ± non-technical skills workshop	High
Praderelli 2016 [57]	USA	Qualitative interview	21	Academic surgeons varied specialities—many in leadership positions, e.g. program directors	Large academic institution	Leadership development programme	High
Ramjeeawon 2020 [58]	UK	Quantitative pre-post test study including non-technical skills scale	16	General and vascular surgeons	Unclear	Simulation ± non-technical skills workshop	High
Sinclair 2015 [59]	UK and ROI	Mixed methods survey with open-ended questions	565	Trainees of all grades and specialties	National survey varied settings	Mentorship	High
Somasundram 2018 [60]	UK	Mixed methods NOTTS scores and feedback form with open-ended questions	48	Urology trainees	Unclear	Simulation + non-technical skills wardrounds	High
Stephens 2018 [61]	USA	Quantitative survey	288	Cardiothoracic surgery residents	Unclear	Mentoring	High
Vitous 2019 [62]	USA	Qualitative interviews	14	Range of clinical areas including general, vascular, plastic, thoracic, and transplant surgery	The University of Michigan	Leadership development programme	High
Vu 2020 [63]	USA	Qualitative- interview	18	General surgery residents	Tertiary care academic institution	Feedback	High
Yule 2015 [64]	USA	Quantitative randomised trial including non-technical skills scale	16	Senior surgical residents	Academic medical center	Simulation ± non-technical skills feedback	Medium

### CMOCs and programme theory

**Fig. 2:**
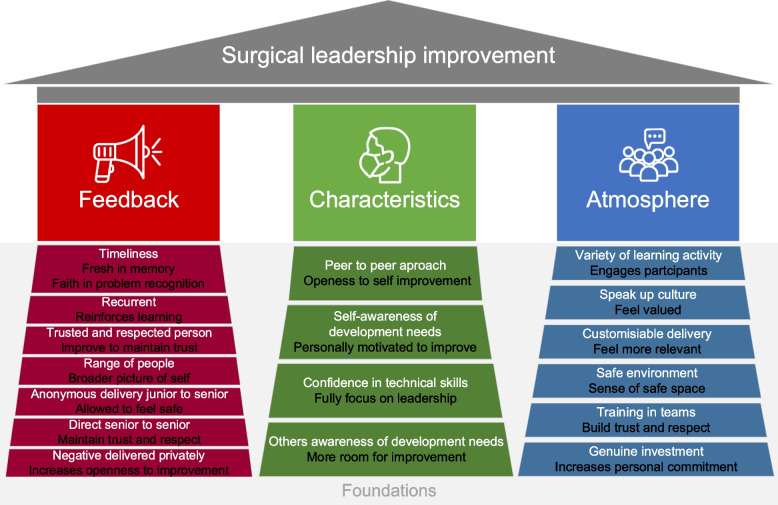
Final programme theory. The Foundational Model of Surgical Leadership Improvement

Individual leadership skills influenced by interventions were grouped into four categories (see Table 4) and Table 5 provides an overview of the 19 CMOCs we identified through our analysis of all the included articles. Our final programme theory, which encompasses all CMOCs, is provided in Fig. 2. Across the 19 CMOCs, the outcome is the same: improved leadership by an individual as defined in the study (see Table 4 for definitions by study), however context and mechanisms differ. The CMOCs have been grouped into three core areas which improve leadership, those which focus on (1) feedback and how feedback is delivered to those partaking in leadership development, (2) the characteristics of the person or people undergoing leadership development, and finally (3) atmosphere, which represents the physical and psychological environment in which leadership development takes place. We now describe each of our 19 CMOCs in turn, with examples from the evidence provided.

**Table 4:** Outcomes of leadership interventions grouped into cognitive skills, interpersonal skills, business skills, and strategic skills

Leadership skill category	Leadership intervention type	Relevant citation in the results
Cognitive skills	• (Situation) monitoring	Nicksa, 2015 [54]; Lee, 2021 [51]
	• Situation awareness	Yule, 2015 [64]; Al-Jundi, 2016 [33]; Ahmed, 2019 [32]; Gostlow, 2017 [40]; Doumouras, 2017 [38]; Nicksa, 2015 [54])
	• Attention to the strengths and weaknesses of colleagues	Vitous, 2019 [62]
	• Self-awareness	Pradarelli, 2016 [57]
	• Self-empowerment	Pradarelli, 2016 [57]
Interpersonal skills	• Communication	Dominguez, 2021 [37]; Yule, 2015 [64]; Nurudeen, 2015 [55]; Lee, 2021 [51]; Ahmed, 2019 [32]; Nicksa, 2015 [54]; Al-Jundi, 2016 [33]; GOSTLOW, 2017 [40]; Doumouras, 2017 [38]; Hu, 2020 [45]; Pena, 2015 [56]
	• Teamwork	Yule, 2015 [64]; Nicksa, 2015 [54]; Gostlow, 2017 [40]
	• Listening skills	Vitous, 2019 [62]
	• Team-building skills	Pradarelli, 2016 [57]
	• Ability to delegate	Vitous, 2019 [62]
	• Encouraging behaviour	Gregory, 2018 [42]
	• Leadership	Pena, 2015 [56]; Yule, 2015 [64]; Lee, 2021 [51]; Nicksa, 2015 [54]; Doumouras, 2017 [38]
	• Participatory approach	Vitous, 2019 [62]
Business skills	• Culture of diversity	Vitous, 2019 [62]
	• Career advancement/progression/development/choice	Kaderli, 2015 [49]; Kawase, 2016 [50]; Lee et al., 2020 [52]; Sinclair, 2015 [59]; Cochran, 2019 [36]; Brook, 2020 [35], Hart 2020 [43]; Stephens, 2018 [61]
	• Professionalism	Nurudeen, 2015 [55]
	• Resource utilisation	Doumouras, 2017 [38]
Strategic skills	• Coordination	Lee, 2021 [51]
	• Decision-making	Yule, 2015 [64]; Hill, 2018 [44]; Ahmed, 2019 [32]; Nicksa, 2015 [54]
	• Problem solving	Nicksa, 2015 [54]
	• Coping with pressure	Gostlow, 2017 [40]

**Table 5:** Context-mechanism-outcome configurations with example quotes

Feedback	
Timeliness of feedback	
CMO1	Leadership interventions are successful in improving leadership (O) if feedback is delivered timely (C) as this makes feedback more relevant and focused as it is fresh in the memory (M)
Vu, 2020 [63]	“Formal written feedback was often received in a delayed fashion, limiting its usefulness to help residents change their behavior in a timely fashion”
Somasundram, 2018 [60]	“In addition, immediate critique from consultant urologists and freeze-frames to discuss performances were also highlighted as useful for the participants’ learning”
Stakeholder	“Feedback is most effective if it is done in real-time rather than a week later or two weeks later.”
CMO2	Leadership interventions are successful in improving leadership (O), if feedback are being provided in a timely fashion (C) as this increases surgeons’ faith that their problems recognised and prioritised (M)
Brindle, 2018 [34]	“Feedback on the issues raised in the debrief was commonly regarded by the clinical leaders as the cornerstone to successful debriefing” “Providing caregivers with early and meaningful feedback on identified problems gave the participants the satisfaction of seeing their problems recognized and prioritized, allowed repetitive issues to be addressed and gave the participants faith that the de- brief achieved a purpose.”
Stakeholder	“For example, in the theatre environment it is much better if members of staff are speaking up early as this mean you can address them early before they become a real problem.”
Recurrent feedback	
CMO3. Leadership interventions are successful in improving leadership (O) if feedback is delivered multiple times (C) as this reinforces learning (M)	
Gregory et al., 2018 [42]	“Conduct a short follow-up survey of the same raters within 3 to 6 months of the baseline to provide feedback about any behavioral changes to reinforce improvement.”
Vu, 2020 [63]	“Formal feedback often omitted any mention of a resident’s leadership performance and was too infrequent to be useful.”
Sinclair, 2015 [59]	“The ideal frequency of mentoring sessions is monthly, although flexibility is required.”
Hart, 2020 [43]	“There was a significant relationship (*p* < 0.0001) between satisfaction with mentorship and frequency of meetings with a mentor, as well as with how mentors were selected (assigned versus found on their own). Those who found their own mentor and met annually reported the lowest average mentor satisfaction (26%), while those who met every 6 months or more reported an average satisfaction of ≥ 80%, regardless of whether their mentor was self-identified or assigned. Residents who met weekly or monthly with their mentors reported 100% satisfaction regardless of how the mentor was selected.”
Stakeholder	“A snapshot is that – a snapshot. Over time, if you spend more time with people – it is very hard to maintain a façade indefinitely. If you ever meet someone once and then you never see them again – it is easy. Multisource feedback should happen over time-dependent process as opposed to one-off-judgement.” “Feedback has to be delivered multiple times not only because it reinforces learning but I think it encourages trust and it improves the whole processes, communication, trust, team building all of that I think. Cannot happen just as a one-off snapshot.” “I have been on quite a few leadership courses now and even the one that […} The (……) programme – I think what helped with that is that initially it was just a one off – two day course and that’s it – you never see them again – I think it is of less value than over a period of time with the same group of people that you go to,engage with more people are much more – find it much easier to open up and talk freely after the first few sessions cause we got to know each other (….)
Feedback delivery by a trusted, respected person	
CMO4. Interventions are successful in improving leadership skills (O) if feedback is delivered by a trusted, respected person (C) as this makes participants want to improve to maintain the trust and respect (M)	
Pradarelli, 2016 [57]	“Some of the coaches with less experience tended to not be able to “make an impression, [and were] too generic.” In contrast, surgeons who had more experienced and reputable coaches found the coaching sessions “insightful and … nice to have [an] objective person to go over 360 [evaluations].” Having a thoughtful, outside perspective provided a “reality check” for participating surgeons.”
Gregory et al. 2018 [42]	“Use a trained mentor or coach to review results, address any pushback, and set leadership excellence goals”
Vu, 2020 [63]	“One resident pointed out, “You’d want to pick people very carefully. Having people...involved where it’s going to feel like any feedback you get is very much constructive and not intended to make you feel bad about anything.” Second, the mentor could provide a valuable third-party perspective to corroborate or reflect on comments from evaluations. As one resident noted, “You could have the opportunity to sit down with someone...who sees everyone’s results and can say, ‘Here’s how you seem to be standing among your peers.”
Stakeholder	“I think by trusting and respecting something I think there’s almost this sort of need or feeling that you actually want to try and accomplish something partly because you want sort of positive feedback from the person that you trust.” “If a trusted colleague of mine said oh actually I think this is an issue or giving feedback I am more likely to take that seriously because I trust and value that individual’s judgement and relationship more than someone who I have never met before.”
Feedback from a range of trusted and respected people	
CMO5. Leadership can be improved if (O) feedback and insights are delivered from a range of trusted and respected people (C) as this provides a broader picture of yourself (M)	
Gregory et al., 2018 [42]	“Balance the rater selection process by inviting both participants as well as their leaders to select from a large pool of potential raters to help ensure both comprehensive and diverse feedback perspectives”
Vu et al. 2020 [63]	“On the whole, residents desired a formalized system to collect and deliver feedback on their leadership performance, especially from multiple sources including junior residents, advanced practice providers, and nurses.”
Cochran, 2019 [36]	All participants described the need for multiple mentors across time and professional roles, providing an overarching them Thirteen participants described a process of developing an intricate and complex system of multiple advisors rather than having 1 mentor who provided all necessary career guidance across the professional lifespan. Participants sought multiple mentors, each with a particular area of expertise, who could provide direction, for example, to their unique phase of career development, discipline and institution, and specific clinical and scholarly needs
Kaderli, 2015 [49]	“In the present study, each mentee had a mean of 1.7 mentors, which has been described as an asset in several publications.”
Stakeholder	“In a providing feedback for how someone working for example, then multisource feedback can be helpful cause you see people in different sources in a different range of environment – and that may show you how people behave in a different way -a trainee may behave and act in a different way with me whereas they behave in a terrible way with others, e.g. the porter or nurse a secretary because of whatever preconceptions that they may have. People to see that difference and getting feedback from different people is important.” “Different mentors at different stages or professional life might be beneficial.”
Delivery of anonymous feedback from juniors to seniors	
CMO6. Leadership interventions are successful in improving leadership (O) if feedback is delivered anonymously from juniors to seniors (C) as this allows juniors to give honest feedback as they feel safe (M)	
Gregory et al., 2018 [42]	“Provide anonymity protections for raters’ anecdotal comments by grouping them into behavioral themes as well as to help leaders to focus more on feedback content rather than its source”
Stakeholder	“In a situation where a more junior is providing feedback to someone more senior they may feel intimidated about saying what they thought.” “I think it would be accepted that juniors give feedback anonymously to more seniors (..) If I was a junior giving feedback to a senior I think I would rather want it to be anonymised – and that is just purely because of that mismatch in the relationship.”
Delivery of direct feedback from a peer or someone more senior	
CMO7. Having feedback from your ranks or from someone more senior (C) is more effective in improving leadership (O) if it is delivered directly as this makes surgeon want to improve to maintain the trust and respect (M)	
Stakeholder	“Having only recently finished being a trainee, I would have valued feedback from my mentors and my supervisors rather than having it come from an anonymous source.”
Negative feedback delivered privately	
CMO8. Leadership interventions are successful in improving leadership (O) if accurate negative feedback is delivered privately (C) as this increases the openness to self-improvement (M)	
Mutabdzic, 2015 [53]	“Some surgeons suggested they would be more comfortable reviewing videos of their surgical procedures with a coach in private, rather than being critiqued in the OR.”
Dominguez, 2021 [37]	“One should definitely exalt the good things publicly and correct privately …. One should not take the resident to the morbidity and mortality meeting to crucify him/her …. When one punishes him/her publicly, the resident will depart from his/her learning curve. If he/she is underperforming, their performance will become worse. If they avoid complex cases, then he/she will avoid more cases to prevent punishment.”
Stakeholder	“I would agree that where possible feedback, especially negative feedback should be delivered on a one-to-one individual basis – I think giving negative feedback in front of other people is – I am go so far to say unprofessional – I think that undermined that individual especially in front of other colleagues – I think there are so many repercussions to saying that I think it can – it causes that person to just withdraw. Now there may be times where feedback needs to be given very quickly and there is no time to do it privately etc. etc. but in large I think negative feedback should be say to do it in an appropriate time – not delay it but do it in a private setting.”
Characteristics	
Peer to peer approach	
CMO9. Leadership interventions are successful in improving leadership (O) if a peer-to-peer approach is taken (C) as this increases an openness to self-improvement (M)	
Greenberg, 2018 [41]	“During implementation, we discovered that some of the concepts and terminology that were adopted from the professional coaching literature needed refinement. The concepts of ‘‘encouraging’’ and ‘‘motivating’’ were somewhat abstract for surgeons. Terms such as ‘‘developing’’ and ‘‘guiding’’ also seemed to reinforce a hierarchical relationship rather than a peer-to-peer relationship. Our initial training focused on these abstract concepts and lacked concrete tactics and approaches that our novice surgical coaches required. We therefore adapted our process and changed the activities of ‘‘encourage/ motivate’’ and ‘‘develop/guide’’ to inquiry, constructive feedback, and action planning.”
Stakeholder	“Even in competitive environments, peer to peer can motivate individuals to improve.” “When it comes to intervention you need to take away as far as you can the hierarchy because if there is a self-composed mismatch in the relationship, I think you are less likely to get openness in terms of the person you are teaching on the leadership courses being able to open up.”
Self-awareness of the need for leadership skills	
CMO10. Interventions are more successful in improving leadership (O) of surgeons when they are bare self aware of needing leadership skills (C) (e.g., beginning a new senior role) as they are more motivated and the intervention has great personal relevance (M)	
Jaffe, 2016 [47]	“Many participants felt that the timing of the program was particularly opportune, as they were at critical junctures in their careers where it was becoming necessary to take on leadership roles to continue to grow.” “This is a key time in my career. Where do I go next?” Another reported that “to get to the next level, I need more skills.”
Stakeholder	“As you get more senior in the organisation you have a greater awareness for leadership skills” “I think there is a stage of career in which leadership intervention is most effective. If you think to improve your leadership you need to have the disposition to do it and you need to have the credibility and legitimacy to get things done. To be relatively senior, but if you are too senior you are probably used to the status quo too much, you are not driving the sort of change that leadership tends to influence.“ “I think they can relate to that leadership intervention more closely because they are imagining themselves more..that they are gonna be doing this particular role sooner rather than later.”
Having confidence in technical skills	
CMO11. Interventions are more successful in improving leadership (O) of those with more confidence in their technical skills (C) as they can fully focus on leadership skills (M)	
Nicksa, 2015 [54]	“Our PGY 2 residents showed improvements on the NOTECHS surveys between the first and second halves of the academic year, correlating to more simulation sessions and more experience going into the simulations. They were able to take more away from it because their focus was on the whole experience rather than just their lone experience within the simulation. In contrast, PGY 1 residents did not demonstrate a significant improvement in their simulation scores over the year.”
Jaffe, 2016 [47]	“To get to the next level, I need more skills.” “[The program] times well for where I’m at. [It’s] time for me to have a clear avenue about what I want to do, create my own road.” “[The] timing is good for my roles as a… fetal program leader… higher leadership things need to be engaged.”
Stakeholder	“If you are more self-confidence and capable technically in terms of technical skills you may have more space to think about leadership.” “If your resources were limited or fixed I think you would be better of giving it to those at the end of the training and early consultant years.”
Others awareness of development needs	
CMO12. Leadership interventions are successful in improving leadership (O) of those with identified leadership deficits (C) as they have more room for improvement (M)	
Gregory et al., 2018 [42]	“In subgroup analysis, the eight chiefs who scored above average (high LTI) at baseline did not improve at follow-up, but the four chiefs who scored below average (low LTI) at baseline had significant improvement after a year of coaching and practice”
J. Hu, 2020 [45]	“LTI (Leadership-teamwork index) scores for surgeons, on average, were lower at baseline and had the greatest on average change compared with primary care and specialists”
Stakeholder	“If they have identified leadership deficits and we have an intervention that is effective that it is expected that they improve when they are insightful of their deficits. Because they have insight and motivation and resource.” “If you start with someone who is poorly on their leadership score, yes, I agree, they are more likely to be able to show an improvement than someone who is already scoring highly on a leadership score.” “I think it is more in my head that there is more room for improvement as opposed to that basically they feel a need to try harder to catch up.”
Atmosphere	
A variety of interactive learning activities	
CMO13. Leadership interventions are effective in improving leadership (O) if they include a variety of interactive learning activities (C) as this engages participants (M)	
Vitous, 2019 [62]	“A lot of these are, you know, sort of business school principles that are not very available to you in medical education, and so it broadens perspectives. And then, when you have a whole group of people broadening their perspectives, the conversation changes.”
Pradarelli et al. 2016 [57]	“The inaugural program curriculum was structured around 4 major domains: leadership, team building, business acumen, and health care context.”
Jaffe et al. 2016 [47]	“Participants felt that in order to fully comprehend the demands on a leader in this healthcare system, they needed sound foundation in the economic forces and business aspects that influence hospitals. One participant referred to his interest in leadership training as obtaining “mini-MBA.” Another reported wanting to “build a strategy for [a hospital setting].” A few participants recognized that “technical” business skills were what they needed most, admitting that they were frequently given financial statements and other documents but did not understand how to analyze or use them for decision making” “Another major desire for participants was to learn how to integrate leadership into the greater healthcare context, both at the hospital level and at the larger policy level. One participant mentioned wanting to know more about “how the [Affordable Care Act] will influence surgery.” Others mentioned learning “how to succeed in the [local] environment,” where there are a “great group of people at all levels” at a given hospital that could learn to “[integrate] across departments.”
Hill et al. 2018 [44]	“The curriculum was based on readings from the book "The Founding Fathers on Leadership" by Donald Philips. Senior surgical residents were randomly assigned to lead chapter discussions. Emphasis was placed on the characteristics of famous individuals instrumental in the emergence of our nation, with concrete examples of how these people demonstrated their roles as admirable leaders. Residents were also instructed to relate these skills back to how they could be incorporated into their everyday role as a physician. At the end of each presentation, the audience expressed their interpretations of the chapter to add to the overall educative experience Sessions took place over a three-week time span with five chapters reviewed weekly.”
Stakeholder	“You need a mixed programme. There is a place for didactic teaching [..] but they need to be kept to a minimum and really focus on key principles [..] because I think we all know we switch off lecture after lecture. The leadership course I was on – I got the most out of it when we broke into smaller groups where we were able to discuss, challenge -even ono-to-one, roleplaying, getting up in front of people to act, when it was much more interactive. I think that is very important” “That links to learning styles. Some people like theory, some people are more practically oriented, some people like to read and reflect and even with an individual it is good to do different things. I think this relates to different learning styles.”
Implementation of speak-up culture	
CMO14. Leadership interventions are successful in improving leadership (O) if they implement a speak up culture (C) as this makes participants feel equally valued and provides a sense of engagement (M)	
Brindle, 2018 [34]	“Levelling the playing field allowed all members to recognize and report threats to patient safety and improve communication” “Even if it was a medical student in the room, I would ask the medical student [to speak first in the debrief] and half the time they had no clue what we had done; but sometimes they might have seen some-thing; and then it was on to the next level trainee, moving all the way up in to the most experienced people; and I usually went last.”
Jayasuriya-Illesinghe, 2016 [48]	“Because junior surgeons and nurses would not speak up and/or raise any concerns they may have with the senior surgeons, there was potential for communication barriers between team members.”
Stakeholder	“An example would be in surgical theatre. I often say if someone sees something that is not right, please say something because […] I am only human – we are all human and ten eyes are better than one…” “The environment should be that as a team every member of the team feels able to say something free to contribute and say something.” “Everyone should be allowed to speak up – end of story. We are all in it together […] Everyone is equally valued in terms of right and responsibility to speak up.” “Where something is not right or could be improved all members of the team should feel that they can contribute and speak up.”
Customised delivery to surgeons’ needs	
CMO15. Leadership interventions are successful in improving leadership (O), if they are customised to surgeons’ needs (C) as this makes the training feel more relevant (M)	
Sinclair, 2015 [59]	“According to respondents, the ideal mentor is one who (…) is chosen by the mentee and has received mentoring training.”
Mutabdzic, 2015 [53]	“This desire for control manifested itself in almost every aspect of the situation including who might be chosen as a coach, the areas of learning that might be addressed, and the decision of how, and even whether, to incorporate the coach’s advice into practice.” “me that would be real coaching where it’s self-identified, I’m motivated, I find the person and then they coach me. Then I decide when I have had enough coaching.”
Pradarelli, 2016 [57]	“Developing a leadership-training program is a personalized and iterative process for individual institutions, and participant feedback is critical to exploring the benefits and weaknesses of the program in detail.”
Stakeholder	“To improve yourself you have to go out of your comfort zone. I think it has to be customised because it then provides insights for your own practice and your organisation’s practice.” “It is gonna be either tailored or required insights from the people providing the intervention for them to reflect and go away and say “ I have learnt this, I have tried this in practice. Unfortunately, it has not worked, do I need to reflect on where I have gone wrong and have that reinforcement with sort of modification to allow for that development for that individuals. Does running the same programme over and over again is likely to improve the outcome.”
Safe learning environment	
CMO16	Intervention are successful in improving leadership (O) if they provide an intimate learning environment (C) as this gives participants a sense of a safe space where they participants speak openly (M)
Sinclair, 2015 [59]	“Ideally, trainees would prefer face-to-face mentoring (94.7%), although email (50.6%) and telephone (30.6%) were also acceptable media. SMS mobile phone messaging (14.7%) and audio/teleconference facilities such as Skype (10.4%) were less popular options.” “Most mentors also wanted to meet face to face (66.8%).”
Foley, 2021 [39]	“Most participants reported that one-on-one coaching in an individual setting would be a useful form of CPD (73.7%, *n* = 87) with only a small proportion disagreeing that this would be beneficial (11.0%, *n* = 13).”
Hill et al., 2018 [44]	“A key component of the course was the comradery amongst the people involved. Our residency program is fairly small and thus each session consisted of only 20 to 30 people. All participants were already very familiar with one another, and this fostered an open learning environment. The willingness to share personal examples of leadership techniques proved to be beneficial for the education of the entire group.”
Somasundram, 2018 [60]	“In later questions, the larger group size was also mentioned as a negative feature; with participants suggesting that they should have been split into smaller groups.”
Stakeholder	“The leadership course I was on – I got the most out of it when we broke into smaller groups where we were able to discuss, challenge -even ono-to-one, roleplaying, getting up in front of people to act, when it was much more interactive.” “Having an opportunity and an environment where people can speak openly without fear of blame or undue criticism – shouldn’t mean that people shouldn’t be challenged – but it should be a non-confrontational kind of way – not being being insulted […] As an example, one of the things that we do now more often is that we review in orthopaedic surgery – we have a weekly X-Ray meeting [….] to review operations that we do […] There is maybe 7 or 8 consultants plus maybe some trainees or fellows and I think this is a good opportunity to share learning and challenge potentially and I think the whole point about this it is a protected space, it is a safe space.”
CMO 17	Intervention are successful in improving leadership (O) if they provide a safe learning environment (C) as this allows interaction with learners which helps them to apply learning to their personal context (M)
Stakeholders	“I think it is also about the ability to individualise your education. In an intimate learning environment you have a higher ratio of teachers to learner and that allows individualisation.” “Allows interaction with learners to help them apply this to their personal context.”
Training in surgical teams	
CMO18. Leadership interventions are effective in improving surgical team leadership (O) if they give surgical teams time to train together and to run through an operation (C) as this help to build trust, rapport, friendship, and mutual respect (M)	
Stakeholder	“One of the things that we don’t do very well in surgical leadership is that we don’t train in teams, we train as individuals and I guess the difference is if you take this into the military. They train as a team together multiple teams and we don’t do this in surgery” “I would say one way to improve leadership in theatre, is for that theatre team should go and spend time practicing how to run through an operation, how to work as a team, developing and ideally continue to keep working in the same group. The problem is in reality, people are pulled, you don’t with the same person and then you wonder why team working and leadership faces such challenge.”
Genuine investment in the intervention	
CMO19. Leadership interventions are effective in improving leadership (O) if there is a genuine investment in the intervention (C) as this is increasing participants’ faith and engagement in the intervention (M)	
Cochran, 2019 [36]	“Interviewees described 3 characteristics of effective mentoring relationships from a mentee perspective: working with a strategic advisor working with an unselfish mentor, and finding a mentor who engages with diverse mentees (in terms of demographics background and academic and clinical interest, different kind of personalities)”
Brindle, 2018 [34]	“At McLeod and Memorial health, this engagement took the form of executive staff being physically present in the operating rooms and other areas where clinical care takes place. This physical presence performed two functions, 1) keeping the executive staff aware and more directly connected with the issues faced by the front-line staff, and 2) reinforcing to the front-line staff the genuine commitment of the institution to the process.”
Ramjeewon, 2020 [58]	“A key strength of the study was the fully-immersive, high fidelity simulation suite which created a realistic environment to attempt to induce the type of behavior seen in the real situation. A fully immersive simulation allows an individual to have a full physical range of motion and interaction with objects and or people. When considering a fully immersive surgical simulation, this would feature a simulated operating theatre and operating theatre team.”
Stakeholder	“If it is investment is sincere people will be able to tell and that does an impact on leadership but if it is not sincere people will disengage” “I think this is one of the biggest problems in certainly the NHS that people have withdrawn or disengaged from this process cause they feel that much of leadership/senior management is a tick-box-exercise…yes they may want to hear you views […] but there is very little done. And if you keep just doing it people just ignore it …and unfortunately that will come down to that particular person and their reputation of whether they act on things that they see or has it always been a show and tell.” “Leaders who truly go round […] and actually do take the time – and that is the problem with sincere investment is it is not a five-minute job – that’s the problem; if you really wanted to learn, the sincere investment does not only need time but also in energy and resources—but often they lack in all three.”

### CMO1-2: timeliness of feedback

The timeliness of feedback was found to be an important contextual feature, which leads to the improvement of leadership. For example, Somasundram et al. (2018) showed that immediate critique from consultants after scenario simulations (referred to as ‘freeze-frames’) were effective for improving participants’ leadership learning [60]. This was echoed by Vu et al., who found that delayed feedback was perceived as limited in its usefulness to residents changing their leadership behaviour [63]. Stakeholders suggested that timely feedback is required because it makes feedback feel relevant and focused, as it is fresh in the memory of the learner. Additionally, a study on debriefing suggested that if feedback on identified problems was provided in a timely manner, surgeons’ faith in the interventions increases, as participants feel satisfied seeing their problems recognised and prioritised [34]. This additional mechanism resonated highly with our stakeholders.

### CMO3: reoccurrence of feedback

Evidence suggests that for feedback to improve leadership, it needs to be provided more than once [42, 43, 59, 63]. For example, studies on feedback interventions identified that follow-up feedback should be provided in the form of a survey within 3–6 months [42]. In support of this, Vu et al. (2020) stated that only frequent feedback can lead to behaviour change [63]. A study on mentoring found that those who had weekly, or monthly mentor meetings were most satisfied with their mentoring arrangements [43]. Since mentors provide feedback we felt that this was supporting the other studies. According to Gregory et al. repeated feedback reinforces leadership improvement overtime [42]. After deliberation with stakeholders, “reinforcement of learning” was agreed as the mechanism.

### CMO4: feedback delivery by a trusted, respected person

Several studies suggest that feedback delivery through a trusted, respected person is an important context for improving the leadership of surgeons [42, 57, 63]. Several studies mentioned the need for an objective person to deliver feedback [57]. or a trained mentor or coach as a suitable and preferred person to provide feedback to surgeons [42, 63]. However, when discussing this with our stakeholders, they concluded that the most important aspect of a person delivering feedback is that you trust and respect them, as this reciprocal relationship makes you want to improve and maintain that person’s trust and respect. We then noted that excerpts revealed that coaches need to be “more experienced or reputable” (Pradarelly, 2016), indicating that trust and respect are key when delivering feedback.

### CMO5: feedback from a range of trusted and respected people

We found that obtaining feedback from a range of people, for example from junior residents, advanced practitioners and nurses [63], is crucial in improving leadership and important in the views of surgeons [42, 63]. Gregory et al. state that feedback from a range of people ensures both comprehensive and diverse feedback. When exploring studies which investigated how mentoring can advance leadership, we found that having multiple mentors appears important in improving leadership for surgeons [36, 49]. However, we found no explicit explanation in the mentorship literature as to why multiple mentors is effective. Stakeholders agreed that obtaining feedback from a range of people can be helpful as it provides a broader picture of yourself. However, stakeholders emphasised that feedback is only helpful in improving leadership if it comes from trusted and respected people. We adapted the CMO to reflect stakeholders’ considerations.

### CMO6: delivery of anonymous feedback from juniors to seniors

Gregory et al. [42] found that anonymous feedback helped improve leadership as it allowed surgeons to focus more on feedback content, rather than its source. Stakeholders felt that anonymous feedback had a role to play but only in the context of juniors providing feedback to seniors. Stakeholders felt that this would allow juniors to provide honest feedback as they feel safe to speak their mind. We have specified the context and mechanisms accordingly.

### CMO7: delivery of direct feedback from a peer or someone more senior

As outlined in the previous CMO, stakeholders felt that feedback was most effective in improving leadership if it was provided directly (rather than anonymously) from a peer or someone more senior. They reasoned that if feedback is provided from someone at your level or above, (i.e. consultant to consultant) you would want to maintain their trust and working relationship and therefore, improve your leadership skills.

### CMO8-9: openness to self-improvement

Our stakeholders suggested that openness to self-improvement is an important mechanism in several contexts. In line with this, two studies indicated that negative feedback is delivered best in a private context [37, 53]. Essentially, surgeons and trainees feel that it is important that they are not challenged or humiliated in front of their peers or colleagues. The private context seems to increase an openness to self-improvement via leadership. In contrast, those who were criticised in front of peers, for example of their handling of surgical cases/at a trauma meeting, were less willing to take on similar cases again in the future or speak openly in meetings because of how it made them feel. In a study on mentoring, we discovered peer-to-peer approaches were more likely to positively impact leadership development [41]. We found that peer-to-peer communication tended to use non-hierarchical language which may have facilitated positive reciprocal reactions to leadership development and a sense of openness between participants. Our stakeholders felt as though the same mechanism (feeling open to and recognising the importance of self-improvement) may be at work here.

### CMO10: awareness for the need for leadership skills

The timing in professional career was an important context for improving leadership. Jaffe et al. [47] describe that leadership training was more effective at point of transition, where it was becoming necessary for surgeons to take on leadership roles to continue to progress, for example when surgeons were moving into a surgeon consultant or surgical director role. Stakeholders confirmed that those who are aware that they need leadership capabilities may feel and be more motivated to improve as the intervention is perceived as more relevant for them. This was particularly poignant when surgeons felt that they lagged behind their peers in this regard.

### CMO11: having confidence in technical skills

Evidence suggests that leadership interventions are more effective in improving leadership of those with more confidence in their technical skills [47, 54]. Surgeons with more confidence in their technical surgical skills were able to focus on their leadership abilities in simulation training. Those with less confidence in their technical skills were facing the dual challenge of focusing on both technical surgical skills and leadership skill development [54]. The timing of leadership development appears relevant to effectiveness, with those surgeons with more confidence in their technical skills perhaps being more likely to benefit from leadership interventions. Stakeholders agreed with this CMO.

### CMO12: having identified leadership deficits

We found evidence to suggest that those who were identified as having leadership deficits, via feedback interventions, demonstrated more improvement in leadership compared to their competent colleagues [42, 45]. Stakeholders suggested that those with identified deficits have more room to improvement and may be more motivated to improve, again elements of peer comparison were mentioned as important.

### CMO13: a variety of interactive learning activities

Studies evaluating leadership courses found that variation in the component leadership learning activities was important for improving leadership [44, 47, 57, 62]. Vitous et al. indicate that broad learning activities, such as team building, business acumen, and self-awareness, expand surgeons’ perspectives. Learning activities included business school principles, leadership in the healthcare context, self-empowerment, and economic forces such as understanding financial statements [47, 57, 62]. We found that active reading, reflection and discussion appeared to be learning activities which improved leadership development specifically [44]. Stakeholders agreed that a variety of learning activities are important but stressed that they needed to be interactive to engage participants.

### CMO14: implementation of speak-up culture

Brindle (2018) found that if all members of a surgical team were allowed to speak-up, about errors for example, this led to an improvement in communication and improved sense of collective leadership [34]. In support of this, Jayasuriya-Illensghe et al. (2016) found that if junior surgeons and nurses are not encouraged to speak up this leads to communication breakdown between surgical teams [48]. Stakeholders agreed that a speak-up culture was a highly important context, whether that be speaking up about unacceptable behaviour style or technical errors. Stakeholders felt that the mechanism at work was “feeling equally valued and a sense of engagement”. Therefore, highlights the importance of considering the organisational culture in which leadership development takes place.

### CMO15: customisation to surgeons’ needs

Evidence from the literature suggests that leadership interventions are effective in improving surgeons’ leadership skills if they are customised to individual surgeons’ needs [53, 57, 59]. For example, studies showed that mentoring and coaching were more effective where surgeons were able to self-select their mentor [53, 59]. A study of a leadership development programme found that intervention effectiveness was dependent on whether the content was personalised to participants and considered their feedback [57]. Mutabzic et al. indicated that ‘sense of control’ over leadership development was the reason why customisation was deemed important in the design of leadership interventions [53]. However, our stakeholders felt that it was less about a ‘sense of control’ but more about the sense of relevance if interventions are customised, and surgeons or surgical trainees had a say in choosing what they felt was most important to them. We therefore adapted this CMO mechanism.

### CMO16-17: safe learning environment

Evidence suggests that interventions improve leadership if they occur in a more intimate learning environment, meaning interventions delivered in person and in small groups or one-to-one. For example, mentoring studies showed that surgeons preferred one-on-one and face-to-face meetings, rather than larger group sessions [39, 59]. Similarly, studies of leadership courses and simulation training indicated that small group learning was preferred by participants [44, 60]. According to Hill et al. intimate learning environments increase participants’ willingness to share personal examples, which may encourage and reinforce their learning as they are actively engaging in the subject matter [44]. Our stakeholders reflected that these environments create a sense of ‘safe space’ where surgeons can speak openly to colleagues. Stakeholders also felt it allows participants to apply the learning to their personal context. We felt that both mechanisms were plausible and recognised both.

### CMO18: training in surgical teams

Stakeholders stated that it would be important for surgical teams to be given time to train in leadership together and to run through operations together to reinforce learning in practice (the opportunity required in AMO theory). Our stakeholders stressed that leadership is a process and only through training together could you build trust, rapport, friendship, and mutual respect leading to surgical team leadership.

### CMO19: genuine investment in the intervention

The concept of ‘genuine investment’ was important for leadership development. We found that if surgeons deem an intervention important in context, and delivered for ‘the right reasons’, then it was more likely to be successful in impacting leadership [34, 36]. For example, mentors who were perceived to be unselfish, and who did not show any tangible benefits from offering mentoring, appeared to positively impact mentee’s leadership development [36]. We found when executive staff were present in the operating room, in a supportive capacity, this signalled to the surgical team members a genuine investment in the leadership intervention [34]. Ramjeewon et al. (2020) affirmed that genuine investment in terms of provision of a realistic setting in simulation studies led to improvement in leadership [58]. This rang true with our stakeholders who concluded that the genuine investment triggered a sense of faith and engagement in the intervention, and in the people delivering it. This led to increased commitment in the programme, and ultimately improvement in leadership.

## Discussion

Realist review methods were used to review the literature describing interventions and strategies which aim to promote evidence-based leadership in healthcare. We aimed to generate a programme theory to explain in which context and for whom surgical leadership interventions work and why. Thirty-three studies and seven stakeholders contributed towards the development of our programme theory which consists of 19 CMOCs. Our findings suggest that surgical leadership interventions improve leadership when feedback is delivered in a timely manner, on multiple occasions and by a range of trusted and respected people. With regard to negative or more developmental feedback, we identified that it is best provided privately. Feedback from seniors to juniors or peers should be delivered directly, whereas feedback from juniors to seniors should be provided anonymously.

While numerous systematic reviews have described the effectiveness of individual leadership interventions in healthcare settings [14–18, 65], we are only aware of one realist review which aimed to understand and explore context and mechanisms; however, this included all medical and surgical specialties [15]. De Brún’s and Auliffe’s review found that training in teams is important for leadership intervention success, suggesting that [it engenders] “the development of a shared understanding and appreciation of skills of others” [15]. A mechanism we further conceptualise narrowly as trust, rapport, friendship, and mutual respect which we also found enables leadership to flourish. De Brún described “open and inclusive communication,” as important [15]. We identified this as a speak-up culture. However, our analysis and consultation with stakeholders suggest that this speak-up culture triggers “feeling equally valued and a sense of engagement” which creates an opportunity for effective leadership to develop [15].

As we anticipated, most of the leadership interventions identified in our review targeted the leadership development of individual surgeons. Many studies in our review report surgeons learning leadership skills through standalone interventions, for example in the studies by Pradarelli et al. and Ramjeeawon et al., but there was very little evidence to describe how and whether this skill-based approach to learning extends into clinical practice [57, 58]. Training surgeons in leadership will only get us so far in making improvements. It is comparable to learning surgery using a textbook, but not letting surgical trainees into the operating theatre to practice and hone their skills in real environments, interacting with team members seniors and patients.

This individualised focus reflects the proliferation of clinical leadership programmes and courses targeted at medical professionals across the globe [3–6]. For example, many papers describe leadership development via nontechnical skills training. These high profile 1-day courses aim to optimise and enhance the performance of individual surgeons [29], yet the evidence for their effectiveness is limited to attendees or peers self-reports of changes in leadership skills [44, 57]. For example, authors asked surgeons ‘do you believe you are a better leader’ and surgeons often replied positively. This provides little sense of the surgeons’ actual capacity and capacity to enact effective leadership. Where leadership improvement was measured objectively, evidence of improvement was captured using tools such as 360° feedback reports, or surgery specific scales including the Nontechnical Skills for Surgeons (NOTSS) and Oxford Nontechnical Skills (NOTECHS) assessment or the Team Strategies and Tools to Enhance Performance and Patient Safety (TeamSTEPPS) scoring. Most studies included in our review did not have a longitudinal design or include multiple sources of evaluation for example, multi-method case study. Hence, it is not clear what impact or for how long any impact of leadership interventions is sustained, let alone whether it can be scaled.

Some might argue that more rigorous approaches to the measurement and evaluation of leadership interventions are required. Firstly, to overcome the apparent responder bias in the literature and secondly, to move a step closer to determining whether the large investment the healthcare sector makes in developing leaders is delivering a return. Yet, we recognise that studies which have sought to establish links between leadership and performance have long been criticised as circumstantial or an anecdotal [66]. Indeed, the causal link have been characterised as an 'act of faith' rather than an empirically proven fact [67]. In part, this is due to a poor conception of what leadership is, and what leadership is not, in public services such as healthcare [67, 68]. There are also methodological problems, such as compounding variables (increased funding for the NHS during the Blair Labour Government resulted in an improved performance effect in the NHS not necessarily attributable to the influence of leadership), and the effect of time lag between leadership actions and their effect that is difficult to discern [69]. Hence, we might best consider ‘effective’ leadership as that aligning with ideal type skill-based models as set out in literature, such as a transformational variant, but one that encompasses an individualistic and distributed configuration of leadership influence, rather than one that focuses upon a ‘heroic’ individual [70].

Nevertheless, the interventions and strategies shown to be most effective in our review include those which aim to raise awareness of the importance of leadership for surgical practice, those that attract people with established confidence in their technical surgical skills, and interventions aimed at surgeons who have been labelled with leadership deficits. Our findings suggest that perceived openness to leadership development, whether that be because surgeons’ have identified deficits or because their technical surgical skills are becoming more innate, was motivational for leadership development. Therefore, leadership interventions can build abilities and capabilities and give surgeons time to focus on their leadership, and an opportunity to practice leadership in the context of clinical practice.

The timing of leadership interventions in surgical careers seems to be a key aspect to their effectiveness. Traditionally, the literature on the timing for the implementation of innovations and behavioural change interventions has focused on discrete events such as triggers to Acton [71, 72]. However, the concept of timing in our review reflected timing relative to a surgical career trajectory, i.e. when mastery of basic and or advanced technical surgical skills have been achieved in earlier years and surgeons have mental capacity to develop in other areas. Our expert stakeholders confirmed that the demands of surgical training in the early stages means that surgeons and surgical teams often have no capacity for non-technical developments. One surgeon described that when training juniors in theatre, patient safety is a first, and that leadership development is not a priority. This highlights specifically the need to practice leadership development in the context of surgery, not in classrooms. The evidence suggests that interventions delivered at key transition points in surgical careers may be more beneficial to surgeons and teams when they have the capacity to undertake additional learning and development.

We found that for interventions to improve leadership in surgery, they need to be delivered in contexts where there is an intimate learning environment within a speak up culture, provide a variety of interactive learning activities, show a genuine investment in the intervention, and be customised to surgeons’ needs. Therefore, leadership of surgical teams may be best developed by allowing mixed discipline surgical teams to train together, rather than training distinct professional groups in isolation, which was the case in most of the literature we reviewed. This was reinforced by expert stakeholders who emphasised that healthcare leadership is rarely enacted in isolation or in distinct professional groups.

## Strengths and limitations

This review represents the first use of realist review methodology to explore how surgical leadership interventions need to be designed to improve the leadership of surgeons, their teams, and trainees. The strengths of the study stem from adopting rigorous methodological guidance for realist reviews as described in the RAMESES quality standards [21] (see Additional file 1). Use of a realist approach has allowed us to place emphasis on how contexts influence outcomes and to focus on identifying generative mechanisms, thereby producing findings that are transferrable across different types of surgical leadership interventions. Whilst we have followed the realist review method and documented the steps that we took to arrive at our programme theory, we are fully aware that (in common with other qualitative research) this method is subjective, iterative, and interpretive, involving many more people than the core review team.

Our study limitations lie in the topic under consideration. The leadership literature is extensive (to crudely illustrate this, there were 330,583 hits for the term ‘leadership’ in MEDLINE at the time of searching). For this reason, we did not include articles which focused on distal outcomes outlined in our initial programme theory (e.g. patient outcomes, or organisational change) [26]. This decision was ratified through discussions with the wider research team and expert stakeholders who concluded that improvements to patient safety may result from advancing leadership but would only ever be considered as indirect evidence.

Our decision to limit distal outcomes was not just a pragmatic choice to prevent being overwhelmed by literature, but a methodological one. The further we extend outcomes, the less confident we can be that the cause can be attributed to the leadership intervention. Organisational and patient level outcomes are likely more affected by a complex set of variables. Hence, our design to limit our study to outcomes that are proximate to the setting in which leadership intervention took place, the surgical unit. Nevertheless, our decision to reduce our focus had consequences for the review as it limited our ability to fully achieve our initial aim, which was to understand how surgical interventions work and generating a strong evidence base to support use of surgical interventions. We may have excluded effective leadership interventions due to their setting, for example interventions delivered at a national scale, such as those programmes offered by the NHS Leadership Academy in the UK [73]. That being said, we recognise that studies which have sought to establish causal links between leadership and performance—whether through improved patient outcomes or organisational change—have been criticised as circumstantial or anecdotal [66]. Having completed the realist review, it would be futile to argue against this narrative. We are in a stronger position to demonstrate the nonlinear relationships between leadership interventions and leadership improvement in surgery. Our programme theory highlights the complexity of the conceptualisations of leadership identified in our review. Our theory is inherently complex and indirect. In developing this work, we have produced evidence which highlights the limitations of expecting to see a causal relationship between the implementation of a leadership intervention—and service improvement; the approach often adopt by the NHS and outlined in the NHS Long Term Plan [4].

In secondary research, the resulting synthesis is only as good as the primary data on which the synthesis is built. Whilst most included studies were of medium and high quality, a major limitation we encountered in our review was that most primary studies included only insight into the impact of surgical leadership interventions on individual leadership in isolation and did not consider the impact on the team or wider department. Because of this limitation in the literature scope, we adopted an individual skills-based framework to summarise the results in Table 4. Whilst this framework facilitates simple presentation of the results, it highlights a wider problem of how narrowly leadership is often conceptualised in surgery. We also found that primary studies often did not provide enough interventional detail to determine fidelity and understand how different aspects of leadership interventions influenced which outcomes and why.

In some cases, our CMOCs had gaps that could not be filled via the literature; most notably, this included a lack of mechanisms evidenced in the literature. This is a not uncommon when conducting realist reviews, and something faced by Price and colleagues in their review of patient safety [28]. They found mechanisms were underdeveloped in the literature. In that research they investigated the processes of meetings and e-mail exchanges to verify and explicate mechanisms with their stakeholder group. A strength of our review is the extent to which we consulted professional and academic experts in this field to review and refine and fill the gaps of our CMOCs.

Ideally, we would have conducted the stakeholder consultations in person as this can help build trust and rapport between participants. However, doing the consultations online was crucial to ensure surgeons could attend and fit the consultations around their work. Further, we would have liked to have consulted a more diverse range of stakeholders, and we acknowledge, for example, that we did not have any female surgeons or early-stage surgical trainees (e.g., training years 1–7) sharing their views. We acknowledge that doing this may have led to changes in the CMOCs. We did invite a range of surgeons and surgical trainees to our stakeholder events, but many declined due to availability.

### Future research directions

It is clear that effective leadership can be important for the surgical profession, but it is by no means a panacea for success. An overarching finding of our review was the lack of literature which examined the entire surgical profession (i.e. not just the individual surgeon) and leadership in surgery more generally, i.e. not confined to an operating theatre. Most literature we identified positioned the surgeon as the target of the intervention and very few studies described the mechanism through which leadership was be improved. This finding is evidence to support additional qualitative research which seeks to explore and illuminate mechanisms, to unpack the ‘black box’ that is leadership improvement.

Whilst it is in important to understand what works in surgical leadership, we also need to understand the changing context in which leadership plays out, both in and outside of the operating theatre and beyond into the hospital and wider surgical community. We suggest that it is the enactment of leadership in context which will become important for improving leadership. Therefore, future research should consider surgical leaders embedded within surgery teams as a unit of analysis. We found that most leadership interventions are not grounded in theory, or evidence. For example, we suggest that the AMO (ability, motivation, and opportunity) theory may be well suited to design interventions which improve surgeons’ performance in the context of practice [11]. We therefore, recommend that academics and clinicians developing, testing and implementing leadership interventions in practice, adopt our programme theory as evidence of what works in surgery, and that researchers perform primary studies which extend our programme theory and further refine our CMOCs.

## Conclusions

In healthcare, evidence-based practice reigns. Yet, when it comes to leadership development in surgery, the same approach to building and adopting the evidence-base in practice falters. Investment in leadership development in healthcare is substantial. To see a return on this investment we need to ensure that the interventions we implement in practice to improve the leadership of surgeons and surgical teams are evidence based and theoretically informed.

Our realist review identified 19 CMOCs which are the starting point for this evidence base. We used the CMOCs to develop the first programme theory to explain in which context and for whom leadership interventions in surgery work and why. The programme theory provides evidence-based guidance for those who are conducting research on leadership in surgery or who are planning or designing evidence-based leadership interventions in surgery.

## Supplementary Information

**Additional file 1.**

## Data Availability

Not applicable.

## Abbreviations

AMO: Ability, Motivation & Opportunity
CMOC(s): Context-Mechanism-Outcome-Configuration(s)
DHSC: Department for Health and Social Care
NHS: National Health Service
RAMESES: Realist and Meta-narrative Evidence Syntheses: Evolving Standards
USD: United States Dollar
